# Case Report: Novel mutations in *TBC1D24* are associated with autosomal dominant tonic-clonic and myoclonic epilepsy and recessive Parkinsonism, psychosis, and intellectual disability

**DOI:** 10.12688/f1000research.10588.1

**Published:** 2017-04-24

**Authors:** Erika Banuelos, Keri Ramsey, Newell Belnap, Malavika Krishnan, Chris Balak, Szabolcs Szelinger, Ashley L. Siniard, Megan Russell, Ryan Richholt, Matt De Both, Ignazio Piras, Marcus Naymik, Ana M. Claasen, Sampathkumar Rangasamy, Matthew J. Huentelman, David W. Craig, Philippe M. Campeau, Vinodh Narayanan, Isabelle Schrauwen

**Affiliations:** 1 Center for Rare Childhood Disorders, Translational Genomics Research Institute, Phoenix, AZ, 85004, USA; 2 Neurogenomics Division, Translational Genomics Research Institute, Phoenix, AZ, 85004, USA; 3 Department of Pediatrics, CHU Sainte-Justine Research Center and University of Montreal, Montreal, QC, H3T 1C5, Canada

**Keywords:** Autosomal dominant Epilepsy, Parkinsonism, psychosis, intellectual disability, TBC1D24

## Abstract

Mutations disrupting presynaptic **protein TBC1D24 are associated with a variable neurological phenotype, including DOORS syndrome, myoclonic epilepsy, early-infantile epileptic encephalopathy, and non-syndromic hearing loss. In this report, we describe a family segregating autosomal dominant epilepsy, and a 37-year-old Caucasian female with a severe neurological phenotype including epilepsy, Parkinsonism, psychosis, visual and auditory hallucinations, gait ataxia and intellectual disability. Whole exome sequencing revealed two missense mutations in the *TBC1D24* gene segregating within this family (c.1078C>T; p.Arg360Cys and c.404C>T; p.Pro135Leu). The female proband who presents with a severe neurological phenotype carries both of these mutations in a compound heterozygous state. The p.Pro135Leu variant, however, is present in the proband’s mother and sibling as well, and is consistent with an autosomal dominant pattern linked to tonic-clonic and myoclonic epilepsy. In conclusion, we describe a single family in which *TBC1D24* mutations cause expanded dominant and recessive phenotypes. In addition, we discuss and highlight that some variants in *TBC1D24* might cause a dominant susceptibility to epilepsy

## Introduction

Mutations in the *TBC1D24* gene are the cause of multiple rare disorders whose phenotype consists of varying degrees of intellectual disability, deafness, cortical malformations, and/or epilepsy ^1^. To date, the disorders caused by TBC1D24 dysfunction make up a continuum of six distinct phenotypes that include DOORS syndrome (Deafness, Onochydystrophy, Osteodystrophy, mental Retardation and Seizures; autosomal recessive; AR), familial infantile myoclonic epilepsy (FIME; AR), progressive myoclonus epilepsy (PME; AR), early-infantile epileptic encephalopathy (EIEE16; AR), autosomal recessive non-syndromic hearing loss (DFNB86; AR), and autosomal dominant non-syndromic hearing loss (DFNA65; AD). *TBC1D24* is highly expressed in the brain and can bind ADP ribosylation factor (ARF) 6, a small GTP-binding protein whose function serves to regulate vesicular trafficking ^2^. Drosophila with mutations in the *sky* gene ( *TBC1D24* orthologue) have a larger readily releasable pool of synaptic vesicles and show a dramatic increase in basal neurotransmitter release ^3^. Overall, evidence demonstrates that TBC1D24 is a critical player in synaptic vesicle endocytosis, neurotransmitter release and presynaptic function ^4^.

In this report, we describe a single family in which *TBC1D24* mutations cause both dominant and recessive phenotypes: dominant tonic-clonic and myoclonic epilepsy, and a recessive severe disorder with epilepsy, Parkinsonian tremor, intellectual disability and psychosis. We discuss and highlight for the first time that dominant inheritance of *TBC1D24* mutations might be associated with epilepsy.

## Case presentation

A 37-year-old Caucasian female (II:1; Figure 1A) with a complex neurological phenotype characterized by myoclonic epilepsy, cerebellar ataxia, cognitive limitation, fatigue, Parkinsonism, photo-sensitivity and psychosis was referred to TGen’s Center for Rare Childhood Disorders ( Supplementary Figure 1).

**Figure 1.**
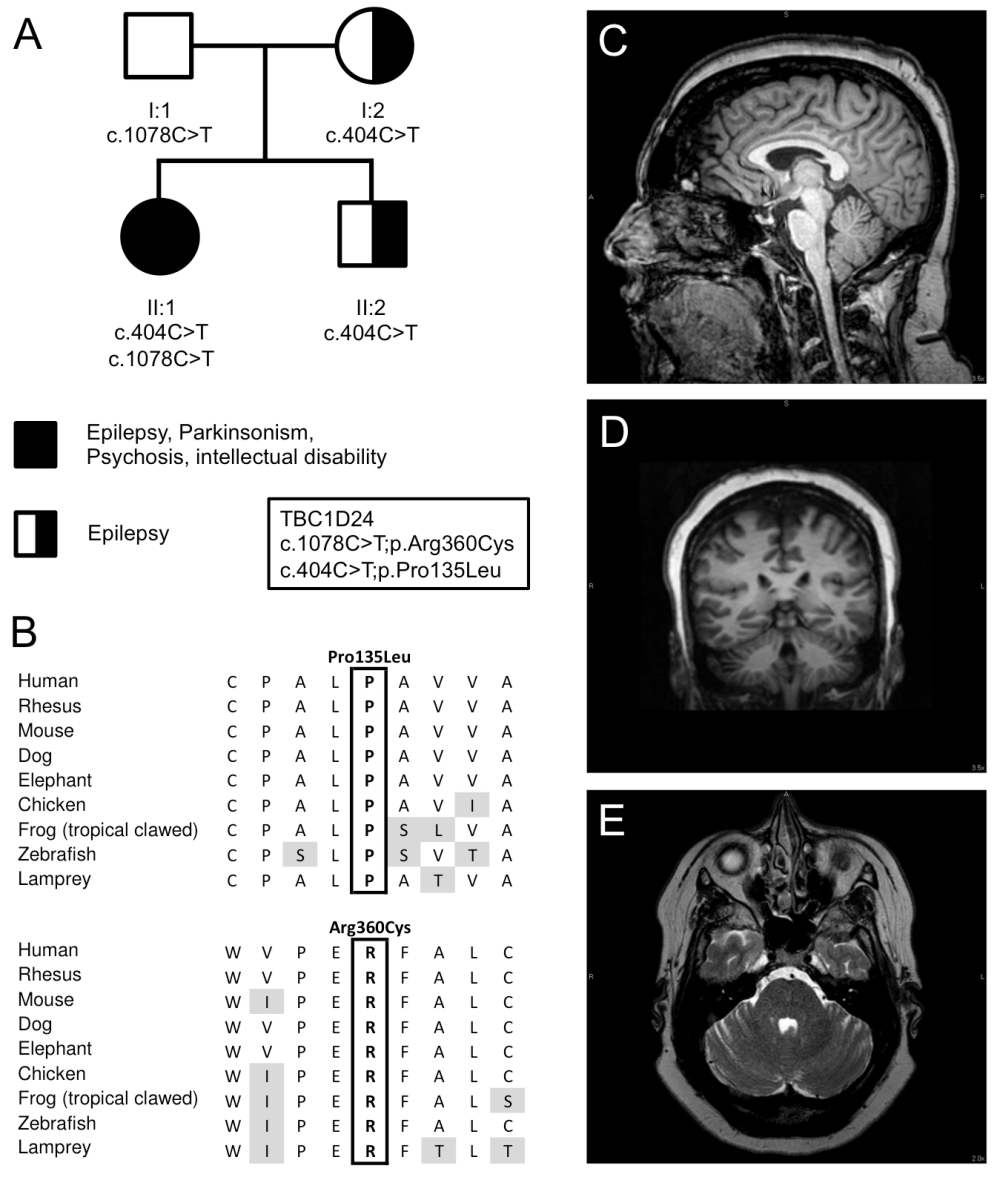
Genetic imaging data. ( **A**) Pedigree and inheritance of the variants in *TBC1D24*, p.Pro135Leu; c.404C>T in exon 2 and p.Arg360Cys; c.1078C>T in exon 4 (NM_001199107.1). ( **B**) Amino acid sequence alignment of both variants between species demonstrating that both amino acids are conserved between species. ( **C**) T1-weighted sagittal MRI scan of brain, illustrating mild cerebellar atrophy affecting the superior vermis. ( **D**) T2-weighted axial MRI scan of the brain showing mild atrophy of the hemispheres. ( **E**) T1-weighted coronal MRI scan of the brain showing mild atrophy of the hemispheres.

The patient is the oldest child of a non-consanguineous marriage, born full term by vaginal delivery. At three months of age, she had daily episodes of vomiting, flexing and extending her legs and shaking her arms lasting 30 minutes to 3 hours. At seven months, she developed a Parkinsonian-like tremor in both hands. She experienced focal episodes that included dystonic attacks: spasm of one side of the body, neck, or on one side of her face, in which she was fully conscious.

At 11 months, the patient experienced generalized seizures. At two years, the patient was able to walk, but did so with an unsteady, wide-based gait. She also experienced an upper extremity myoclonus with dystonic features. A neurological examination revealed ptosis, limited upward gaze, and left esotropia. At 2.5 years, a cranial CT scan suggested mild anterior cerebellar atrophy.

The patient’s current symptoms include severe fatigue and an unpredictable sleep cycle. Her speech was delayed (2.5 years onset) and is slow and slightly dysarthric. She was alert and oriented to person and place, but her attention and concentration were reduced. Pure-tone audiometry at 250–8,000 Hz at age 15 showed that she had normal hearing (0–15 db), and tympanometry showed normal stapedial reflexes. She continuously complained of intermittent tinnitus. An ophthalmologic exam at age 13 and 36 showed she had a reduced visual acuity (ETDRS and Snellen acuity testing; OD:40, Snellen 20/160; OS: 36; Snellen 20/200), and optical coherence tomography at age 36 showed a significant retinal and optical nerve thinning (central macular thickness 191 OD, 205 OS, and average nerve rim thickness 71 OD, 67 OS). Intermittent horizontal nystagmus was also observed. Phalanges and nail beds were normal. A physical examination showed that the patient’s muscle tone, strength, and deep tendon reflexes were normal. Although she was able to walk, she had a broad based ataxia (37-years old; Supplementary Video 1). She had dysmetria on nose to finger test, as well as a coarse action tremor. She continues to have involuntary jerking of upper more than lower extremities that last up to an hour. The patient has not had any focal or generalized seizures since the age of 35.

Her cognitive ability was impaired, with a significant decline observed during childhood but no significant change in cognitive ability since adolescence. At age 4.5, a Wechsler Intelligence Scale for Children (WISC-R) test showed an average overall IQ of 93, and at age 8, this was 81 ^5^. A WISC-III test at 14 years resulted in an IQ score of 65, and at the age of 32 she scored an overall IQ of 67 measured by the Wechsler Adult Intelligence Scale - IV (WAIS-IV) ^6, 7^. Neuropsychological exams at age 14 and 32 showed severe impairment in the area of visual scanning, working memory, and executive function. She was administered the trail making test and stroop test at the age of 14 and 32 and showed similar scores ^8, 9^: For the trail making test, she took 119 seconds to complete trail A as compared to 102 seconds in at age 14. She was unable to complete the trail B task in both occasions. Both of these suggest severe impairment in the area of visual scanning, working memory, and set shifting. The stroop test of executive functioning revealed a severely impaired range on both occasions (age 14 and 32; T < 20). Some variability was seen in the area of working memory. The patient previously performed in the average range on the digit span (age 15), but scored in the mild deficit range on more current testing (age 32). However, on the arithmetic test, which is also a measure of working memory, she previously performed in the severe deficit range, but currently scored in the moderate deficit range. Performance on measures of perceptual reasoning, processing speed and verbal abstraction were impaired as well, as measured by WISC-III and WAIS-IV at age 14 and 32, and remained stable between both ages.

Since 12 years of age, the patient has had episodes of visual and auditory hallucinations. In addition, she suffered from paranoia, agitated behavior, disinhibition and depression. Overall, the patient presents with intellectual disability with increasing symptoms of psychosis.

The patient has undergone two muscle biopsies, and showed little evidence for mitochondrial disease. An increased complex I and IV activity were noted using electron transport studies, although muscle and mitochondrial morphology were normal. Multiple tests for mitochondrial and nuclear DNA mutations were normal. A magnetic resonance imaging (MRI) test in 2014 showed mild cerebellar atrophy in the superior cerebellar vermis and the cerebellar hemispheres ( Figure 1C–D), and an overnight video EEG showed diffuse mild background slowing consistent with mild cerebral dysfunction, but no epileptiform activity was observed during the test.

The patient’s family history ( Figure 1A) is notable in that the mother (I:2) experienced general tonic-clonic seizures before the age of 5, but was not treated with anticonvulsants. The mother also noted that her father had similar episodes. The mother also reported the presence of hearing loss starting at the age of 40, and needed hearing aids in both ears by the age of 60. Moreover, the patient’s older brother (II:2) experienced general myoclonic spells similar to that of the patient and was treated with anticonvulsants through the age of 18.

## Methods

### Sequencing

DNA was extracted from the blood of both parents and proband. Exomic libraries were prepared with the SureSelect All Human XT v5 exome kits (Agilent Technologies, Santa Clara, CA, USA), following the manufacturer’s protocol. Sequencing was performed by 101bp paired-end sequencing on a HiSeq2000 instrument (Illumina Inc, San Diego, CA, USA). Filtered reads were aligned to the Human genome (Hg19/GRC37) using the Burrows-Wheeler transform (BWA-MEM; v0.7.8). Reads where sorted and polymerase chain reaction (PCR) duplicates were removed using Picard (v1.111) and base quality recalibration, indel realignment were performed using the Genome Analysis Toolkit (GATK; v3.1-1). Variants were called jointly with HaplotypeCaller and recalibrated with GATK, annotated with dbNSFP (v2.9) and snpEff (3.5h) for protein-coding events. Prediction scores were loaded from dbNSFP (v2.9) and used for filtering.

Sanger sequencing was performed on the proband and brother by GeneDx (Gaithersburg, MD, USA), and on the parents by the authors. In short, the target areas of the gene were PCR amplified and capillary sequencing was performed. A bi-directional sequence was assembled, aligned to reference gene sequences based on human genome build GRCh37/UCSC hg19 and analyzed for known familial sequence variant(s) (Applied Biosystems Inc., Foster City, CA, USA).

## Results

Exome sequencing in the proband and parents led to the identification of compound heterozygote mutations in *TBC1D24* in the patient (II:1), p.Pro135Leu; c.404C>T in exon 2 and p.Arg360Cys; c.1078C>T in exon 4 (NM_001199107.1) ( Figure 1). These mutations were confirmed by Sanger sequencing in the entire pedigree ( Figure 1). Neither variant was observed in the ExAC Browser database of 60,706 unrelated individuals ^10^, and both were predicted to be damaging by Polyphen 2, MutationTaster, have a high combined annotation dependent depletion (CADD) score (26.5 and 17.7 for P135L and R360C respectively) ^11^ and are conserved between species ( Figure 1B). Both the mother and younger brother, affected by seizures at younger ages, are heterozygous for the p.Pro135Leu variant. The father is carrier of the p.Arg360Cys variant and asymptomatic ( Figure 1A).

## Discussion

We identified two previously unreported pathogenic variants in the *TBC1D24* gene that segregate in a family with a severe complex neurological disorder and tonic-clonic and myoclonic epilepsy (p.Pro135Leu and p.Arg360Cys; Figure 1A). The p.Pro135Leu missense mutation follows an autosomal dominant pattern associated with mild seizures in the proband, mother and younger brother. The proband shows a severe but atypical recessive *TBC1D24* neurological phenotype and is compound heterozygote for p.Pro135Leu and p.Arg360Cys. The p.Arg360Cys carrier shows no clinical symptoms.

Mutations in *TBC1D24* so far have shown to cause an autosomal recessive variable phenotype, ranging from non-syndromic hearing loss, epileptic disorders, to DOORS syndrome ^1^. An autosomal dominant inheritance pattern has only been described in cases with non-syndromic slowly progressive adult onset hearing impairment ^12^. However, there are other reported cases of *TBC1D24* mutation carriers who had seizures, such as the mother of a child with DOORS syndrome, who carries a frameshift mutation (c.1008delT, p.His336GlnfsTer12*) and who had absence seizures as a child ^13^. In another family with recessive deafness caused by *TBC1D24* mutation, a heterozygous carrier of a missense mutation (c.208G>T, p.Asp70Tyr) had epilepsy since the age of 3 continuing in adulthood (individual IV-8 of pedigree PDKF799 in 14). In another family, the mother and half-sister of an individual carrying the NM_001199107.1:c.32A>G, p.Asp11Gly mutation had epilepsy beginning in adulthood (they were not tested for the mutation ^15^). Finally, the father of a patient with the following *TBC1D24* mutations, c.1460dupA, p.His487Glnfs*71 and c.313T>C, p.Cys105Arg, also had a history of seizures ^1^. While it is difficult to attribute with certainty the epilepsy in these carriers or possible carriers to the *TBC1D24* mutations, the incidence of epilepsy in carriers is higher than what would be expected by a chance co-occurrence, given that there are less than 40 families described thus far and the prevalence of epilepsy in the general population is 7 per 1000 individuals ^16^. Moreover, as suggested with the family we describe here, some variants might be more susceptible to cause dominant epilepsy. With regards to Parkinsonism, it was previously described in one individual with DOORS syndrome who was later found to have two *TBC1D24* mutations (c.619C>T, p.Gln207* and c.1126G>C, p.Gly376Arg, PMID: 27281533) ^1, 17^.

The behavior of the p.Pro135Leu variant is distinct from previous reported variants, and is of interest as it acts almost in a semi- or partial dominant manner. This variant occurs at a highly conserved position in the Rab-GAP N-terminal Tre2–Bub2–Cdc16 (TBC) domain of the protein. It was recently discovered that this domain directly binds phosphoinositides through a cationic pocket and that phosphoinositide binding is critical for presynaptic function ^4^. A fly model with 3 clinically pathogenic mutations (3Glu mutant) in the phosphoinositide-binding pocket causes severe neurological defects, including impaired synaptic-vesicle trafficking and seizures ^4^.

The p.Arg360Cys variant is located between the TBC and TBC–LysM (TLDc) domain. p.Arg360Leu, a change at the same site as p.Arg360Cys, has been described in a recessive state in a patient with progressive myoclonus epilepsy ^18^. Functional studies in primary mouse cortical cells transfected with p.Arg360Leu mutant showed a significant reduced induction of outgrowth in neurite length compared to wild-type ^1^. Both p.Arg360Leu and p.Arg360Cys only seem to exhibit a clinical phenotype in a recessive or compound heterozygote state in this and previous studies ^18^.

In conclusion, this family’s clinical presentation highlights the broad spectrum of both AD and AR *TBC1D24* disorders, including Parkinsonism, psychiatric symptoms, and autosomal dominant tonic-clonic and myoclonic epilepsy.

## Data availability

The data referenced by this article are under copyright with the following copyright statement: Copyright: © 2017 Banuelos E et al.

Exome data of individuals have been added to the Database of Genotypes and Phenotypes (dbGaP; http://www.ncbi.nlm.nih.gov/gap) under project phs000816. Both variants have been reported to ClinVar ( http://www.ncbi.nlm.nih.gov/clinvar/) under variation IDs SCV000494664 (p.Arg360Cys), SCV000494665 and SCV000494666 (p.Pro135Leu; NM_001199107.1). The raw sequence data of the father (C4RCD_0194), mother (C4RCD_0193), and propositus (C4RCD_0192) were submitted to the Sequence Read Archive (SRA; http://www.ncbi.nlm.nih.gov/sra) with the respective Biosample ID numbers SAMN05687268, SAMN05687209 and SAMN05687491.

## Consent

Written informed consent for publication of their clinical details, (identifiable) clinical images and videos was obtained from the legally authorized representative (as the patient has a diminished decision-making capacity, due to her intellectual disability and her disorder described here) and the patient’s family. The study was explained to the extent compatible with the subject’s understanding, and was enrolled into the Center for Rare Childhood Disorders program at the Translational Genomics Research Institute (TGen). The study protocol and consent procedure was approved by the Western Institutional Review Board (study number, 20120789).

## Supplementary material

Supplementary Figure 1: Proband at the age of 37 years.

Supplementary Video 1: A video of the walking at the age of 37 years, showing an unsteady, wide-based gait.
