# Supplementary figures and images for: Case Report: Novel mutations in TBC1D24 are associated with autosomal dominant tonic-clonic and myoclonic epilepsy and recessive Parkinsonism, psychosis, and intellectual disability
Source: F1000Res. 2017 Apr 24;6:553. [Version 1] doi: 10.12688/f1000research.10588.1 (PMC5473401; doi:10.12688/f1000research.10588.1)

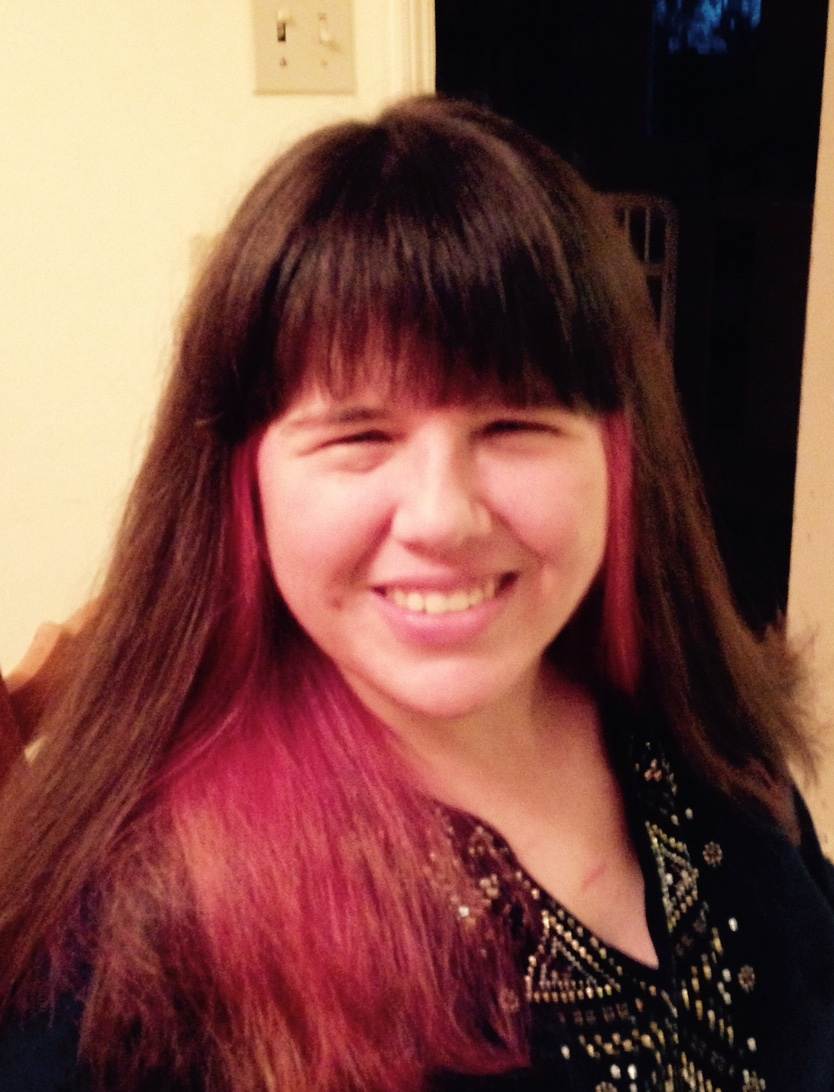

Supplement: Supplementary file 1 [file f1000research-6-11410-s0000.tgz › f528fafa-fa0a-4e96-81e3-17b817468321.tiff]
