# Supplementary material for: Case Report: Novel mutations in TBC1D24 are associated with autosomal dominant tonic-clonic and myoclonic epilepsy and recessive Parkinsonism, psychosis, and intellectual disability
Source: F1000Res. 2017 Apr 24;6:553. [Version 1] doi: 10.12688/f1000research.10588.1 (PMC5473401; doi:10.12688/f1000research.10588.1)
Supplement: Peer review history

# Peer review: Referee response for version 1

**DOI:** 10.5256/f1000research.11410.r23438

**Published:** 2017-06-13

**Authors:** Brian Appavu

**Affiliations:** 1 Columbia University Medical Center, New York, NY, USA

I read this case report with great interest. TBC1D24-related diseases represent a unique subset of conditions for which the rapid emergence of both clinical and basic science data has helped elucidate insight into the pathophysiology of this condition. This case report adds to the existing literature of TBC1D24-related diseases, and suggests a novel inheritance pattern in which an autosomal-dominant pattern of inheritance leads to a phenotype that includes epilepsy. This paper can benefit from minor revisions. 

1. With regard to the case presentation, were any seizures or specific episodes of myoclonus captured on video EEG monitoring? If so, what did they show? If myoclonus was captured, did it appear either cortical or subcortical?
2. The terms, variant and mutation are used at various points throughout the manuscript. To make the terminology clear, I would refer to those mutations that are pathogenic as pathologic variants, rather than mutations.
3. Discussion, paragraph 2 line 1: For grammatical purposes, change the following line to "Mutations in *TBC1D24* so far *have been* shown to cause an autosomal recessive variabe phenotype, ranging from non-syndromic hearing loss to epileptic disorders and DOORS syndrome"
4. Discussion, paragraph 2 line 7: Did epilepsy begin at 3 months or 3 years of age? Be specific.
5. Conclusion: Given that this is the first paper to suggest an an autosomal-dominant mode of inheritance for TBC1D24-related epilepsy, commentary regarding the need for further functional studies of this and other related variants would help strengthen this manuscript.

I have read this submission. I believe that I have an appropriate level of expertise to confirm that it is of an acceptable scientific standard.

# Peer review: Referee response for version 1

**DOI:** 10.5256/f1000research.11410.r22162

**Published:** 2017-05-03

**Authors:** Deepa S. Rajan

**Affiliations:** 1 Division of Child Neurology, Children's Hospital of Pittsburgh, Pittsburgh, PA, USA

Interesting Case. 

1. In the attached video, the phenotype would most likely fit into ataxia/ wide based gait/ cerebellar origin in my opinion and think that the term parkinsonism might be misleading. Other than the mentioned tremor were there any other features to suspect extrapyramidal disease?
2. Were there any other variant of currently unknown significance on the exome?

I have read this submission. I believe that I have an appropriate level of expertise to confirm that it is of an acceptable scientific standard.
